# Gut microbiota and microbiota-derived metabolites promotes endometriosis

**DOI:** 10.1038/s41420-023-01309-0

**Published:** 2023-01-25

**Authors:** Sangappa B. Chadchan, Sumanta K. Naik, Pooja Popli, Chandni Talwar, Satwikreddy Putluri, Chandrasekhar R. Ambati, Michael A. Lint, Andrew L. Kau, Christina L. Stallings, Ramakrishna Kommagani

**Affiliations:** 1 Department of Pathology and Immunology, Baylor College of Medicine, One Baylor Plaza, Houston, TX 77030 USA; 2 Department of Molecular Microbiology, Washington University School of Medicine, St. Louis, MO 63110 USA; 3 Center for Women’s Infectious Disease Research, Washington University School of Medicine, St. Louis, MO 63110 USA; 4 Department of Molecular and Cellular Biology, Baylor College of Medicine, One Baylor Plaza, Houston, TX 77030 USA; 5 Advanced Technology Core, Baylor College of Medicine, One Baylor Plaza, Houston, TX 77030 USA; 6 Division of Allergy and Immunology, Department of Medicine, Washington University School of Medicine, St. Louis, MO 63110 USA; 7 Department of Molecular Virology and Microbiology, Baylor College of Medicine, One Baylor Plaza, Houston, TX 77030 USA

**Keywords:** Infertility, Endocrine reproductive disorders, Metabolomics

## Abstract

Endometriosis is a pathological condition of the female reproductive tract characterized by the existence of endometrium-like tissue at ectopic sites, affecting 10% of women between the age 15 and 49 in the USA. However, currently there is no reliable non-invasive method to detect the presence of endometriosis without surgery and many women find hormonal therapy and surgery as ineffective in avoiding the recurrences. There is a lack of knowledge on the etiology and the factors that contribute to the development of endometriosis. A growing body of recent evidence suggests an association between gut microbiota and endometriosis pathophysiology. However, the direct impact of microbiota and microbiota-derived metabolites on the endometriosis disease progression is largely unknown. To understand the causal role of gut microbiota and endometriosis, we have implemented a novel model using antibiotic-induced microbiota-depleted (MD) mice to investigate the endometriosis disease progression. Interestingly, we found that MD mice showed reduced endometriotic lesion growth and, the transplantation of gut microbiota by oral gavage of feces from mice with endometriosis rescued the endometriotic lesion growth. Additionally, using germ-free donor mice, we indicated that the uterine microbiota is dispensable for endometriotic lesion growth in mice. Furthermore, we showed that gut microbiota modulates immune cell populations in the peritoneum of lesions-bearing mice. Finally, we found a novel signature of microbiota-derived metabolites that were significantly altered in feces of mice with endometriosis. Finally, we found one the altered metabolite, quinic acid promoted the survival of endometriotic epithelial cells in vitro and lesion growth in vivo, suggesting the disease-promoting potential of microbiota-derived metabolites. In summary, these data suggest that gut microbiota and microbiota-derived metabolome contribute to lesion growth in mice, possibly through immune cell adaptations. Of translational significance, these findings will aid in designing non-invasive diagnostics using stool metabolites for endometriosis.

## Introduction

Endometriosis, a chronic disease in which endometrial glands and stroma implant outside the uterus, afflicts 1 in 10 reproductive-age women [1, 2], which accounts for around 196 million women worldwide. The most common symptoms of endometriosis are infertility and pelvic pain [3, 4]. Other symptoms include excessive bleeding and pain with menstruation, intercourse, and bowel movements or urination [5]. A crucial factor in endometriosis is unopposed estrogen signaling and resistance to progesterone. Other factors include altered immune function, epigenetic modifications stimulated by environmental toxicants, and endocrine disrupters [6, 7]. Despite decades of research, current therapies are only limited to either symptomatic pain relief or hormonal therapies or surgical excision of endometriotic lesions that do not prevent recurrences.

The principal theory is that endometriotic lesions establish when endometrial tissue moves retrogradely into the peritoneal space during menstruation and implants on surrounding tissues, such as the intestine or peritoneum [8]. Given that 90% of women experience retrograde menstruation, it is believed that the immune system usually clears these cells. However, in 10% of women, the immune cells are unable to clear the endometrial cells, which then adhere and proliferate to form lesions. These lesions then spread via inflammation due to release of pro-inflammatory cytokines and growth factors in the peritoneal cavity [9, 10]. Studies from endometriosis mouse models found elevated levels of Tumor Necrosis Factor-alpha (TNFα), interleukin 6 (IL-6), Macrophage Inflammatory Protein 1 alpha (MIP-1α), and MIP-2 in peritoneal macrophages [11–14]. Further, TNFα–MMP9 (Matrix metallopeptidase 9) axis generates endometriotic steroid receptor hormone 1 (SRC-1) isoform, which plays a crucial role in endometriosis disease progression in mice [11]. There is also a profound infiltration of neutrophils in ectopic tissue that occurs during early onset of endometriosis and its progression, when neutrophils and macrophage inflammatory proteins MIP-1α and MIP-2 are elevated in peritoneal fluid [12]. Treatment of Interleukin 1 beta (IL-1β) to the cells from endometriotic lesion but not in normal endometriotic cells leads to induction of Vascular endothelial growth factor (*VEGF)* and *IL-6* transcripts. Moreover, inflammasome, a multiprotein complex, stimulates the secretion of IL-1β and IL-8, resulting into the multiple host responses. These studies suggest that enhanced IL-1β signaling, which occurs in response to inflammasome activation, promotes endometriotic angiogenesis [15, 16]. Consistently, the peritoneal fluid of women with endometriosis has elevated IL-1β, which promotes the release of cytokines, such as Interleukin 8 (IL-8), and growth factors that contribute to neovascularization and monocyte chemotaxis in endometriotic explants [17, 18]. This evidence strongly supports the role of inflammatory response in this process, most of which comes from mouse models of endometriosis [11, 12].

Multiple evidence suggests that microbiota is altered in women with endometriosis. First, Chen et al. reported different cervical and uterine microbiome communities in women with and without endometriosis [19]. Second, a study by Shan et al. observed lower alpha diversity of gut microbiota and a higher Firmicutes-to-Bacteroidetes ratio in women with stage 3/4 endometriosis (*n* = 12) than healthy controls (*n* = 12) [20]. Further, Ata et al. found, in a cohort of 14 women with stage 3/4 endometriosis and 14 healthy women, that women with endometriosis had elevated *Gardnerella*, *Streptococcus*, *Escherichia*, *Shigella*, and *Ureoplasma* in their cervix and elevated *Shigella/Escherichia* in their stool [21]. Third, a study by Svensson et al. carried out on human stool samples revealed high alpha (the microbial diversity of a single sample) and beta diversities (a measure of similarity or dissimilarity between two communities), as well as the Firmicutes-to-Bacteroidetes ratio [22] in control group (*n* = 198) than the endometriosis patients (*n* = 66) [22]. Fourth, the human peritoneal microbiome analysis revealed the abundance of *Acidovorax, Devosia, Methylobacterium, Phascolarctobacterium*, and *Streptococcus* in the peritoneal fluid of endometriosis patients than the matched controls [23], Fifth, in a mouse model of endometriosis in which endometrial fragments are injected into the intraperitoneal space, endometriosis was linked with alterations in the gut microbiome [24]. Finally, in our first study, we found that treatment with either broad-spectrum antibiotics or metronidazole after lesion initiation reduced lesion growth in a surgical model of endometriosis [25]. Whereas, in our recent study we revealed that, the microbial metabolite, n-butyrate protects against endometriosis disease progression in mouse model of this disease [26]. Although, these studies provided a correlative relation between gut microbiota and endometriosis, it is not clear whether the gut microbiota directly influences the lesion formation and growth. In this study, we found that depletion of the gut microbiome reduces endometriotic lesion growth and that lesion growth is rescued by orally gavaging the mice with feces from mice with endometriosis. Additionally, using germ-free donor mice, we found that uterine microbiota is not essential for endometriotic lesion growth. Furthermore, we showed that the gut microbiota modulates immune cell populations in the peritoneum of mice with endometriosis. Additionally, our metabolomic analysis revealed a signature of fecal metabolites whose abundance significantly differs between mice with and without endometriosis. If these results are recapitulated in humans, they could benefit in leading new strategies to diagnose and treat endometriosis.

## Results

### Endometriosis lesion growth is reduced in microbiota-depleted mice

In our previous study [25], we performed endometriosis surgery and then provided mice with antibiotics in their drinking water. However, this model prevented us from determining the causal role of gut microbiota in endometriosis disease progression. To address the causal role of microbiota in endometriosis, we considered two possible models: germ-free or microbiota-depleted mice. Germ-free mice generated by surgically delivering pups, sterilizing them, and rearing them in germ-free isolators [27]. Microbiota-depleted (MD) mice are generated by raising mice under standard conditions and then orally gavaging adults with broad-spectrum antibiotics vancomycin (50 mg/kg), neomycin (100 mg/kg), metronidazole (100 mg/kg), and ampicillin (100 mg/kg), plus 1 mg/kg amphotericin-B, an anti-fungal agent included to overcome sporadic overgrowth of *Candida* species) every 12 hours for seven days. The resulting mice have many of the same physiological characteristics as germ-free mice, such as hypoplastic lymphoid tissue and altered gene expression profiles of intestinal epithelial cells [28–31]. However, we preferred the microbiota depletion model over germ-free mice model for several reasons. First, surgically inducing endometriosis in germ-free isolators is extremely challenging and can lead to contamination and infection. Second, germ-free mice have several developmental defects and lack an educated immune system as they are maintained in sterile isolators from birth [32]. Finally, microbiota-depletion enables eliminating microbiota in adult mice and can be re-colonized with gut microbiota with fecal microbiota transfers [33]. Thus, compared to germ-free mice, microbiota-depleted mice are more suitable for our studies [30].

**Fig. 1:**
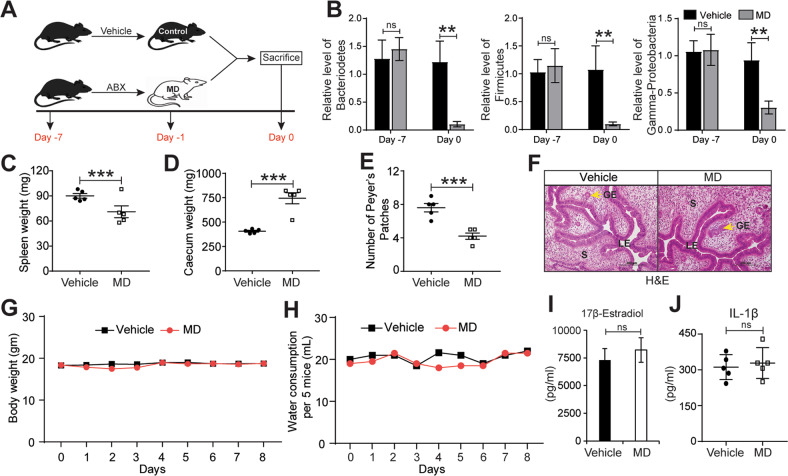
Generation of microbiota-depleted mice using antibiotics. **A** Schematic of experimental timeline and procedures. **B** Quantification of relative abundances of Bacteroidetes, Firmicutes, and Gamma-proteobacteria in feces from vehicle and Microbiota-depleted (MD) mice. **C**-**D** Wet weights of **C** spleen and **D** caecum in indicated treatment groups at a sacrifice. **E** The number of Peyer’s patches from indicated treatment groups. **F** Representative images of Hematoxylin and Eosin-stained uterine cross-sections from the indicated treatment groups. LE, Luminal Epithelium; GE, Glandular Epithelium; S, Stroma. Yellow arrows indicate the gland. **G**-**H** Mouse **G** body weight and **H** water consumption at indicated time points in vehicle and MD mice. **I**-**J** Relative level of **I** 17β-Estradiol in serum and **J** IL-1β in peritoneal fluid of indicated treatment groups. Data are presented as mean ± SE (*n* = 5 mice per group). **P < 0.01, ***P < 0.001, and ns, non-significant.

**Fig. 2:**
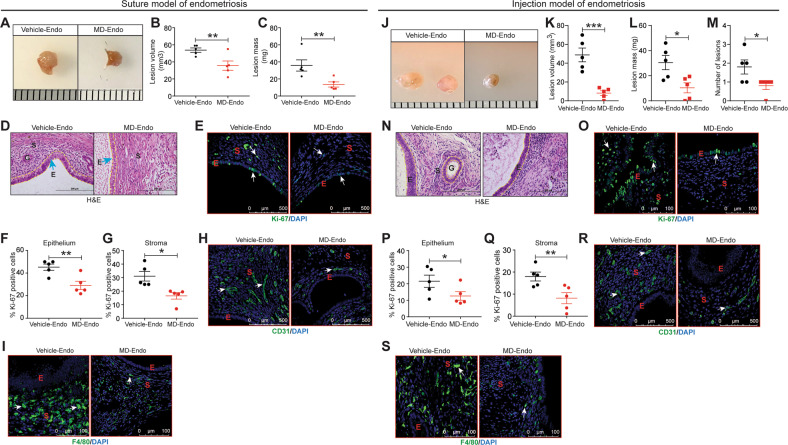
Gut microbiota promotes endometriosis disease progression in mice. **A**, **J** Ectopic endometriotic lesion representative images from **A** suture model **J** injection model. **B**, **K** The endometriotic lesion volumes from **B** suture model and **K** injection model. **C**, **L** The endometriotic lesion masses from **C** suture model and **L** injection model from the indicated groups 21 days postinduction of endometriosis. **M** Number of lesions per mouse in injection model from the indicated groups 21 days post-induction of endometriosis. **D**, **N** Representative images of ectopic lesions from **D** suture model and **N** injection model from the indicated treatment groups stained with Hematoxylin & Eosin (H&E). **E**, **O** Representative images of ectopic lesions from **E** suture model and **O** injection model from the indicated treatment groups stained with anti-Ki-67 antibody. **F**, **P** Percentages of Ki-67-positive cells in endometriotic lesion epithelium, **F** suture model and **P** injection model; **G**, Q stroma, **G** suture model and **Q** injection model. Representative images of ectopic lesions stained with **H**, **R** anti-CD31 in **H** suture model and **R** injection model; **I**, **S** anti-F4/80 **I** suture model and **S** injection model from the indicated treatment groups. White arrows indicate positive cells. Data are presented as mean ± SE (*n* = 5), *P < 0.05, **P < 0.01 and ***P < 0.001.

We used microbiota-depleted mice generated as described above (Fig. 1A) and confirmed by quantitative PCR of feces that they had significantly less Bacteriodetes, Firmicutes, and Gamma-Proteobacteria than control mice (Fig. 1B). Consistent with previous research [28, 29], the microbiota-depleted mice had significantly smaller spleens, larger ceca, and fewer Peyer’s patches than control mice (Fig. 1C-E). Thus, microbiota depletion produced the similar phenotypes that are seen in germ-free mice [30]. Importantly, uteri from microbiota-depleted mice had typical endometrial epithelia, glands, and stroma, indicating that microbiota depletion had no detrimental effect on gross uterine morphology (Fig. 1F). This treatment had no effect on both the body weight or water consumption of the mice (Fig. 1G, H). Finally, we also showed that there was no change in the serum level of 17β-Estradiol (Fig. 1I) or level of IL-1β (Fig. 1J) in the peritoneal fluid of MD mice. Next, we surgically induced endometriosis in both control and MD mice by autologously transplanting a piece of the uterus onto the peritoneum and assaying the resulting lesions 21 days later (Fig. S1A). Lesions in control/vehicle mice were significantly larger and more obviously fluid-filled and vascularized than lesions in microbiota-depleted mice (Fig. 2A-D). Lesions in microbiota-depleted mice contained fewer proliferative cells (Ki-67-positive) (Fig. 2E-G), endothelial cells (CD31-positive) (Fig. 2H), and macrophages (F4/80-positive) than lesions in control mice (Fig. 2I).

In the previous section we evaluated the role of gut microbiota using a suture model of endometriosis. In this model, during the induction of endometriosis, mice underwent a major surgery that may trigger an inflammatory response in the mice. To avoid effects from the surgery, we adopted an injection model, where endometriosis can be induced in mice without performing the surgery. We induced the injection-based endometriosis in both control/vehicle and MD mice and analyzed 21 days after (Fig. S1B). Similar to the surgical/suture model of endometriosis, lesions in vehicle-treated mice in the injection model are also significantly larger and in greater number than the lesions of MD mice (Fig. 2J-M). Additionally, the lesions from control/vehicle-treated mice showed typical endometriosis-like characteristics, which is lacking in the MD mice (Fig. 2N). Like the surgical/suture model, lesions in microbiota-depleted mice from the injection model also contained fewer proliferative cells (Fig. 2O-Q), CD31-positive endothelial cells (Fig. 2R), and macrophages (F4/80-positive) than lesions in control mice (Fig. 2S). Irrespective of the endometriosis model system, these results clearly indicate a role for the microbiota in endometriotic lesion growth.

### Gut microbes are required for endometriotic lesion growth in mice

**Fig. 3:**
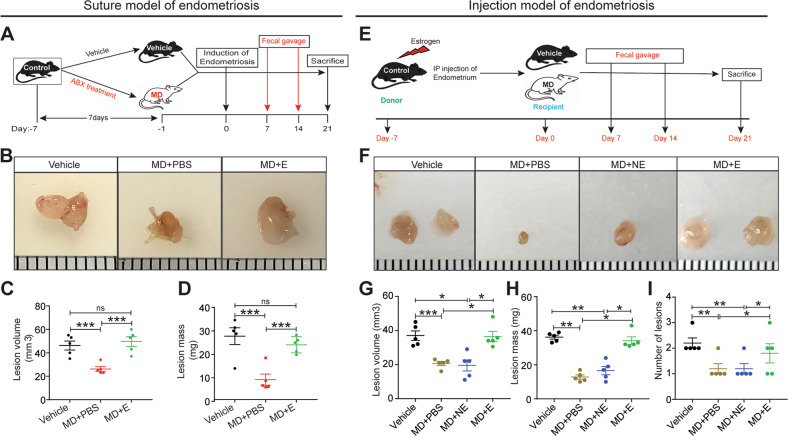
Gut microbiota is required for endometriotic lesion growth in mice. **A**, **E** Schematic of experimental timeline and procedures for **A** suture model and **E** injection model for the fecal microbiota transfer (FMT) experiments. Microbiota-depleted mice underwent endometriosis induction and received an oral gavage of PBS (MD + PBS), feces from mice without endometriosis (MD + NE) or feces from mice with endometriosis (MD + E). **B**, **F** Ectopic endometriotic lesion representative images from **B** suture model and **F** injection model. **C**, **G** Ectopic lesion volumes from **C** suture model and **G** injection model. **D**, **H** Ectopic lesion masses from **D** suture model and **H** injection model from the indicated groups 21 days after the induction of endometriosis. **I** Number of lesions per mouse in injection model from the indicated groups 21 days post-induction of endometriosis. All the indicated data is 21 days after induction of endometriosis. Data are presented as mean ± SE (*n* = 5), *P < 0.05, **P < 0.01, ***P < 0.001, and ns, nonsignificant.

Next, we determined whether gut microbes are required for endometriotic lesion growth. To test this possibility, we treated mice with vehicle (control) or antibiotics (microbiota depletion), surgically induced endometriosis, and then administered either PBS or fecal material from mice with endometriosis through oral gavage (Fig. 3A). Whereas transplantation of fecal material from mice with endometriosis developed typical control-like endometriotic lesions in microbiota-depleted mice, feces from healthy mice failed to restore lesions (Fig. 3B-D and S2A). Subsequently, we performed similar studies in the injection-based mouse model of endometriosis. We injected uterine fragments from control donor mice intra-peritoneally, and then transplanted feces from mice with and without endometriosis by oral gavage (Fig. 3E). Feces from mice without endometriosis (MD + NE) developed significantly smaller and fewer endometriotic lesions than feces from mice with endometriosis (MD + E) (Fig. 3F-I). Additionally, lesions in microbiota-depleted mice that received feces had similar numbers of proliferative epithelial and stromal cells (stained for Ki-67) (Fig. S2B-D), endothelial cells (CD31-positive cells) (Fig. S2E), and macrophages (stained with F4/80) as lesions in control mice (Fig. S2F). These findings indicate that gut microbes are required for endometriotic lesion growth.

### Uterine microbiota might be dispensable for endometriotic lesion growth in mice

**Fig. 4:**
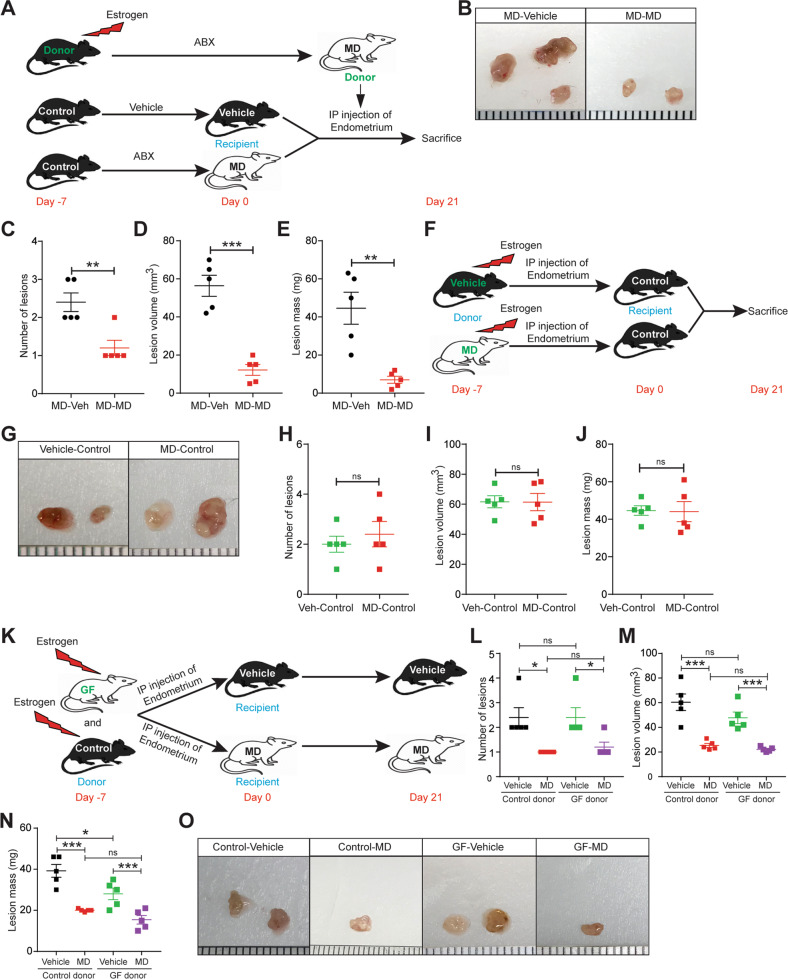
Uterine microbiota might be dispensable for endometriotic lesion growth in mice. **A**, **F**, **K** Schematic of experimental timeline and procedures. Ectopic endometriotic lesion **B**, **G**, **O** representative images, **C**, **H**, **L** number of lesions per mouse, **D**, **I**, **M** volumes and **E**, **J**, **N** masses from the indicated groups 21 days after induction of endometriosis. Data are presented as mean ± SE (*n* = 5), *P < 0.05, **P < 0.01, ***P < 0.001 and ns nonsignificant.

The above results could indicate that microbiota depletion altered the uterine microbiome, causing autologously transplanted uterine fragments in microbiota-depleted mice to be less able to form lesions than fragments in control mice. To test this idea, we injected endometrial fragments from donor MD mice into the peritoneal space of recipient vehicle and MD mice (Fig. 4A). Compared to control mice, recipient MD mice developed fewer and smaller endometriotic lesions (Fig. 4B-E). To further confirm that uterine microbiota is not responsible for the differences in endometriotic lesion growth, we injected endometrial fragments from the vehicle and MD donor mice into the peritoneal space of control mice (Fig. 4F). As expected, mice receiving the endometrial fragments either from vehicle or MD donor, developed endometriotic lesion of similar mass, size, and number. (Fig. 4G-J). Subsequently to strengthen the hypothesis that the uterine microbiota might be dispensable for endometriotic lesion growth in mice, we injected uterine fragments from control or Germ-Free (GF) mice into the control and MD mice (Fig. 4K). Irrespective of the origin of donor uterine fragment, compared to control mice, only MD mice developed smaller and fewer endometriotic lesion (Fig. 4L-O). These findings suggest that uterine microbiota might be dispensable but not the gut microbiota for endometriotic lesion growth in mice.

### Gut microbiota impacts the peritoneal immune cell populations in mice with endometriosis

**Fig. 5:**
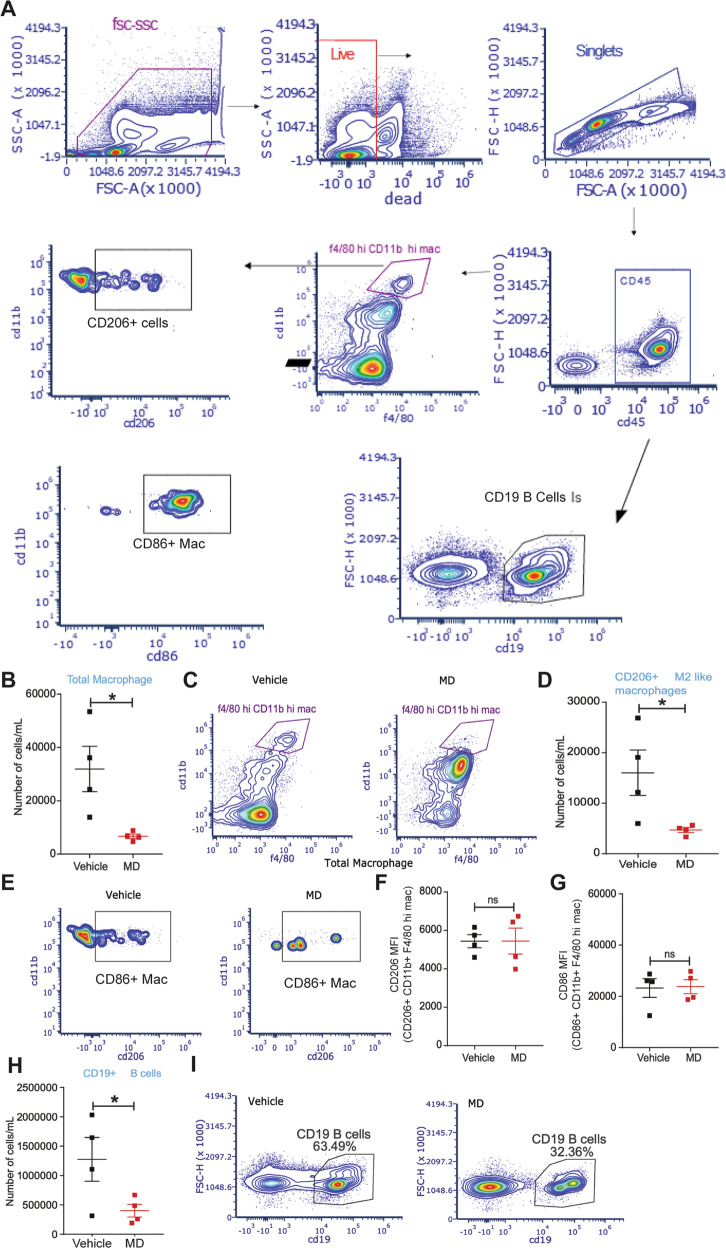
Gut microbiota depletion affects the macrophage and B cell population in the peritoneal fluid of mice with endometriosis. The endometriosis was induced in the vehicle and MD mice as shown in Fig. S1B and flow cytometric analysis was carried out on the peritoneal fluid from mice with endometriosis. **A** Flowchart of flow cytometric plots for the cell sorting using the Cytek Aurora. **B** The relative number of total microphages per mL and **C** flow cytometric plots in the peritoneal fluid from vehicle and MD mice. **D** The relative number of CD206^+^ M2-like macrophages (M2-like mac) per mL and **E** flow cytometric plots in the peritoneal fluid from vehicle and MD mice. **F**, **G** Mean fluorescence intensity of F CD206^+^ CD11b^+^ F4/80 hi mac (M2 like Macrophage) and G CD86^+^ CD11b^+^ F4/80 hi mac (M1like macrophage) in the peritoneal fluid from vehicle and MD mice. **H** The relative number of CD19^+^ B-cells per mL and **I** flow cytometric plots in the peritoneal fluid from vehicle and MD mice. All the indicated data is 21 days after the induction of endometriosis. Data are presented as mean ± SE (*n* = 4), *P < 0.05 and ns nonsignificant.

**Fig. 6:**
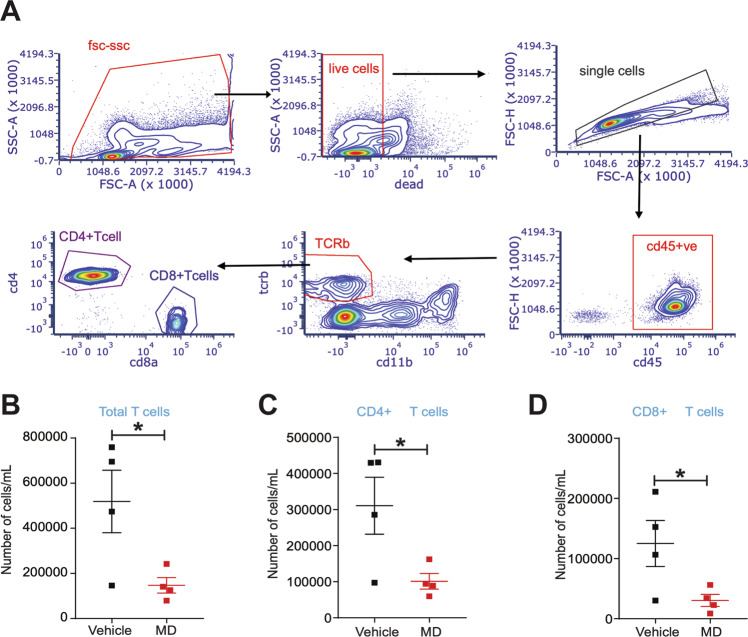
Gut microbiota depletion affects total, CD4^+^ and CD8^+^ T cell population in the peritoneal fluid from mice with endometriosis. **A** Flowchart of Flow cytometric plots for the cell sorting using Cytek. **B**-**D** The relative number of B total T cells per mL, **C** CD4^+^ T cells per mL and **D** CD8^+^ cells per mL in the peritoneal fluid from vehicle and MD mice. The endometriosis was induced in the vehicle and MD mice as shown in Fig. S1B and flow cytometric analysis was carried out on the peritoneal fluid 21 days after the induction of endometriosis. Data are presented as mean ± SE (*n* = 4), *P < 0.05.

It is well documented that inflammation is associated with endometriosis and the gut microbiota are known to modulate the inflammatory milieu of the peritoneum. Thus, we analyzed the immune cell populations in the peritoneal fluid of vehicle and MD mice to determine if altered immune cells composition was associated with reduced lesion growth in MD mice. The flow cytometric sorting strategy is shown in Fig. 5A. Compared to vehicle-treated mice, MD mice contained fewer total (Fig. 5B, C) and CD206^+^ (M2-like macrophage) (Fig. 5D-E) macrophages in the peritoneum. However, the mean fluorescence intensity (MFI) for CD206 on CD206^+^ CD11b^+^ F4/80^hi^ M2-like macrophages (Fig. 5F) and the MFI for CD86 on CD86^+^ CD11b^+^ F4/80^hi^ macrophages (Fig. 5G) was unchanged. We also observed a lower number of CD19^+^ B cells, Total T cells, CD4^+^ T cells, and CD8^+^ T cells in MD mice compared to the vehicle-treated mice (Fig. 5H, I) and (Fig. 6A-D). These results suggest that the gut microbiota impacts endometriotic lesion growth, possibly through the modulation of peritoneal immune cell populations. However, in-depth functional studies with specific immune cell-deficient mouse models will uncover the precise mechanism by which gut microbiota drives peritoneal immune function in endometriosis.

### Fecal metabolite landscape between mice with and without endometriosis

**Fig. 7:**
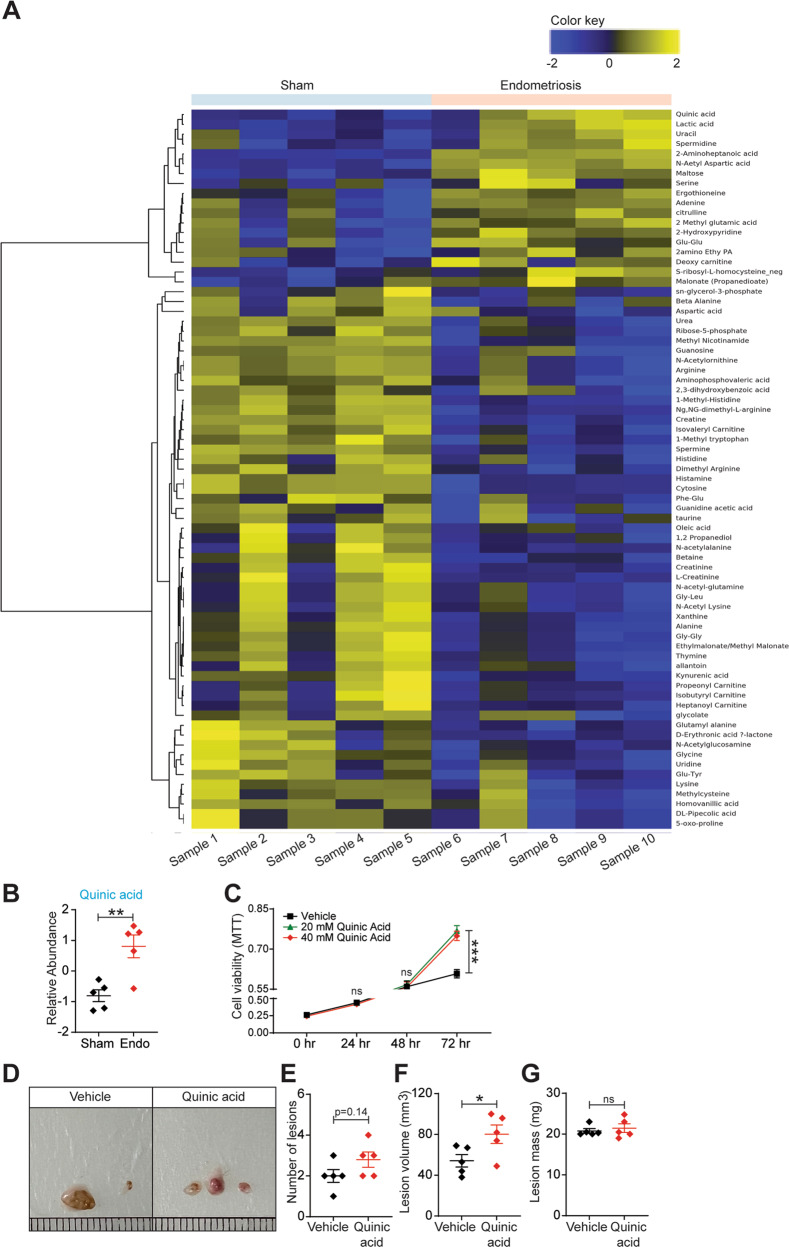
Fecal metabolites differ between mice with and without endometriosis. **A** The heat map depicting the metabolites that were differentially present between mice with and without endometriosis with cutoff of FDR < 0.25. **B** Quinic acid is present at higher level in feces of mice with endometriosis. Stool samples from mice with and without endometriosis were subjected to LC-MS (in the Baylor College of Medicine Metabolomics Core) to detect ~150 water-soluble metabolites. Each row is a metabolite, and each column is a stool sample from an individual mouse; (*n* = 5 mice per group). Data are presented as mean ± SE. **C** MTT cell viability assays of iHEECs/Luc treated with 20 mM and 40 mM Quinic acid for indicated time points. Results are shown as mean ± SE (*n* = 3) and experiment repeated three times. Ectopic endometriotic lesion **D** representative images, **E** number of lesions per mouse, **F** volumes and **G** masses from the vehicle and QA treated groups,14 days after induction of endometriosis. Data are presented as mean ± SE (*n* = 5), *P < 0.05, **P < 0.01, ***P < 0.001 and ns, non-significant.

One possibility by which gut microbiome could affect endometriosis is through gut microbiota-derived metabolites. Interestingly, particular microbiome-derived metabolites are associated with both obesity [34] and autism spectrum disorder [35]. To begin to test the idea that gut-bacteria-derived metabolites influence endometriosis disease progression, we measured the relative metabolites (>150) in feces from mice that underwent endometriosis surgery and those that underwent sham surgery. The results revealed an identification of a signature of (>50) metabolites in feces from sham mice compared to the control mice with endometriosis (Fig. 7A). We plotted the relative abundances of six of these metabolites namely, Quinic acid (QA) (Fig. 7B), Cytosine, 1-Methyl-Histidine, Ng, NG-Dimethyl L-Arginine, 2-Aminoheptanoic acid and N-Acetyl Aspartic acid (Fig. S3A), which were differentially present in feces of mice with endometriosis. We investigated the in vitro effect of these metabolites on cells derived from human endometriotic lesions and found that QA most significantly increased the proliferation of immortalized human endometriotic epithelial cells expressing luciferase (iHEECs/Luc) (Fig. 7C). Whereas, other five metabolites only moderately modulated the iHEECs/Luc proliferation (Fig. S3B-F). Based on these in vitro results, we further studied the in vivo effects of QA on endometriosis lesion growth in mice. After the induction of endometriosis, from day 1, mice were orally gavaged with QA (5 mg/kg) every 24 h for 14 days. The mice that received the QA developed significantly larger endometriotic lesions than mice that consumed the vehicle, whereas we did not observe any significant change in the lesion numbers (Fig. 7D-G). These data indicate that QA promotes lesion growth but not the establishment of lesions. Taken together, our findings suggest a role for gut microbiota and microbiota-derived metabolites in endometriosis disease progression.

## Discussion

In this study, we have provided evidence that gut microbiota plays a pivotal role in endometriosis disease progression in mice. The major finding includes 1) the uterine microbiota might be dispensable for endometriosis disease progression. 2) The fecal microbiota transfer from mice with endometriosis rescues the endometriosis phenotype in both suture, as well as an injection model of endometriosis, suggesting gut bacteria drive disease progression. 3) The antibiotics-mediated depletion of gut microbiota modulates immune cells populations in the peritoneum of mice with endometriosis. 4) Fecal metabolites are altered in mice with and without endometriosis. 5) Treatment of endometriotic cells and mice with Quinic acid significantly enhanced the cellular proliferation and endometriotic lesion growth, respectively.

Recently, a correlation between altered microbiota and endometriosis pathogenesis is reported [20–24, 26, 36]. For example, women with endometriosis are more likely to have uterine microbial dysbiosis than women without endometriosis [19, 21, 37, 38]. Further a study on human stool samples revealed the differences in the alpha and beta diversities and altered Firmicutes-to-Bacteroidetes ratio [22]. A more recent study reported the altered peritoneal microbiome in women with endometriosis [23]. Moreover, several studies including our previous work showed that gut microbial communities are altered in mice as well as women with endometriosis [21, 24, 25, 39]. We found that bacteria in the gut, as opposed to any other site, are required for endometriotic lesion progression. Since women with endometriosis are often susceptible to inflammatory bowel disease, our findings therefore shed light on the potential connection between endometriosis and colonic diseases via gut bacteria [40].

Endometriosis is primarily recognized as an inflammatory disease. Upon ectopic implantation of endometrial fragments, macrophages and neutrophils are first recruited. Activated macrophages predominantly secrete numerous pro-inflammatory cytokines and chemotactic and angiogenic growth factors [41]. A recent study demonstrates that lesion-resident macrophages are derived from eutopic endometrial tissue that infiltrate large peritoneal macrophages and monocytes [42]. Hence, the depletion of eutopic endometrial macrophages results in reduced endometriotic lesion growth. In contrast, constitutive inhibition of monocyte recruitment significantly reduces peritoneal macrophage populations. Strikingly, this results in an increased number of lesions, suggesting a protective origin-specific role of monocyte-derived macrophages in the peritoneal cavity to limit the development of lesions [42]. Further, it is well documented that microbial metabolites act as a messenger between gut microbiota and immune function [43–45]. Given our observation that the microbial metabolites are altered in mice with endometriosis and depletion of gut bacteria reduces the endometriosis-associated inflammation [25] and immune cell population, future efforts could explore the role of particular microbiota or derived metabolite in context to the regulation of endometriosis-associated inflammation.

Previously, the signatures of the metabolome in Gout revealed that the metabolites associated with uric acid excretion, purine metabolism, and inflammatory responses are altered [46]. Further, GS-MS-based analysis of metabolome revealed that 13 metabolites differed between controls and irritable bowel syndrome (IBS) patients [47]. Interestingly, fecal volatile organic compound (VOC) profiling suggested a significant increase in fecal ester compounds in nonalcoholic fatty liver syndrome (NAFLD) patients [48]. These increasing pieces of evidence suggest that the gut microbial dysbiosis and their derived metabolites are associated with multiple pathological conditions. Additionally, arachidonic, and linoleic acid derivatives are associated with several pregnancy-associated pathologies, such as gestational diabetes mellitus and pre-eclampsia. Whereas arachidonic acid metabolite levels are higher in women’s ovarian tissue when suffering from the polycystic ovarian syndrome. These findings suggest the association of metabolites with fertility-related pathological conditions [49]. Serum samples from endometriosis patients exhibit augmented levels of citrate, lactate, 3-hydroxybutyrate, alanine, leucine, valine, threonine, lysine, glycerophosphatidylcholine, succinic acid and 2-hydroxybutyrate whereas levels of lipids, glucose, isoleucine, and arginine are reduced [50, 51]. Further, in another report, two metabolites triacylglycerols and α‐amino acids were found abundant in the serum of endometriosis patients when compared with matched controls [52]. However, we found that 2-aminohepatonic acid, N-Acetyl Aspartic acid; Maltose, Lactic acid, and Quinic acid are significantly upregulated in feces of mice with endometriosis. Treatment of endometriotic cells and mice with QA significantly enhanced the cellular proliferation and endometriotic lesion growth in mice respectively. Interestingly, lactic acid, which was found elevated in the human serum samples from endometriosis patients [51], also increased in the feces of a mouse model of endometriosis in our study. Importantly, quinic acid might prove to be useful as a non-invasive diagnostic tool for the early detection of endometriosis, which is an unmet need for women who suffer from this painful disease.

At present, only limited reports are available on endometriosis and stool metabolomics, and none focused on the relationship between the two. A recent study conducted on feces of mice with endometriosis revealed that Chenodeoxycholic acid and Ursodeoxycholic acid were upregulated whereas, Alpha-linolenic acid and 12, 13s-epoxy-9z, 11, 15z-octadecatrienoic acid (12,13-EOTrE) were downregulated [53]. Additionally, another finding suggests that endometriosis is associated with abnormal lipid metabolism, which is demonstrated with low BMI in humans and reduced body fat stem cells, and disorder of lipid metabolism in the animal model [54, 55]. Based on these previous findings by others and our group [21, 24, 36] in relation to gut microbiota and endometriosis, we speculated that the microbial dysbiosis and difference in metabolites might be in a self-regulation mode, which can provide the necessary adaptive microenvironment for endometriosis establishment.

In summary, our findings provide novel insight into the molecular underpinnings of endometriosis, suggesting that gut microbiota-derived metabolites may be a new important predictive marker for endometriosis. An in-depth study focusing on the specific microbiota or associated metabolites in endometriosis-associated inflammation in the context of human endometriosis patients will be next on our agenda.

## Material and methods

### Animal studies

Mice (C57BL/6, Taconic) were housed in an animal facility at Washington University, School of Medicine, St. Louis, MO, USA or Baylor College of Medicine Houston, TX, USA under standard 12-h light-dark cycle with access to food and water ad libitum. Germ-free mice were maintained in a gnotobiotic facility using flexible plastic isolators and monitored monthly to ensure sterility. The germ-free mice were bred in house for these experiments. All animals were housed 5 animals per cage maximum and monitored daily for welfare. All mice used for the study were between 8 and 10 weeks of age. All animal experiments were approved by the Institutional Animal Care and Use Committee (Protocol #2019-1079 and AN-716).

### Statistical analyses

All statistical analyses were performed using GraphPad Prism 9 software (GraphPad Software, San Diego, USA). All data are presented as means ± SEM. A two-tailed paired Student t-test was used to analyze between-group differences in experiments comparing two experimental groups. Analysis-of-variance (ANOVA) by non-parametric alternatives was applied for comparisons between multiple groups as appropriate.

A detailed description of the materials and methods used in this study is available in the online Supplementary Material.

## Supplementary information

Supplemental file

Figure S1

Figure S2

Figure S3

## Data Availability

We have uploaded the raw metabolomics data, which will be available in the NIH Metabolomics Workbench (National Metabolomic Data Repository) database with the project ID (ST002410).
