# Supplementary material for: Gut microbiota and microbiota-derived metabolites promotes endometriosis
Source: Cell Death Discov. 2023 Jan 25;9:28. doi: 10.1038/s41420-023-01309-0 (PMC9873805; doi:10.1038/s41420-023-01309-0)
Supplement: Supplementary file 1 — Supplemental file [file 41420_2023_1309_MOESM1_ESM.docx]

**Supplementary file for**

**Gut microbiota and microbiota-derived metabolites promotes endometriosis**

Sangappa B. Chadchan^1^, Sumanta K. Naik^5&7^, Pooja Popli^1^, Chandni Talwar^1^, Satwikreddy Putluri^3^, Chandrasekhar R. Ambati^4^, Michael A. Lint^6&7^, Andrew L. Kau^6&7^, Christina L. Stallings^5&7^ and Ramakrishna Kommagani^1&2^*

^1^Department of Pathology and Immunology,

^2^Department of Molecular Virology and Microbiology,

^3^Department of Molecular and Cellular Biology,

^4^Advanced Technology Core,

Baylor College of Medicine, One Baylor Plaza, Houston, TX, 77030, USA.

^5^Department of Molecular Microbiology,

^6^Division of Allergy and Immunology, Department of Medicine,

^7^Center for Women’s Infectious Disease Research,

Washington University School of Medicine, St. Louis, MO, 63110, USA.

***Correspondence to:**

Ramakrishna Kommagani, PhD

Associate Professor

Department of Pathology and Immunology

Department of Molecular Virology and Microbiology

Alkek Center for Metagenomics and Microbiome Research
T228A, One Baylor Plaza

Baylor College of Medicine

Houston, TX, 77030

Email: rama.kommagani@bcm.edu

Phone: (713)-798-5085

**Short title**: Microbiota-derived metabolites and endometriosis.

The authors have declared that no conflict of interest exists.

**This PDF file includes:**

Supplementary Materials and Methods

Supplementary Figure legends

**Supplementary Materials and Methods:**

**Microbiota depletion with antibiotics**: Depletion of gut microbiota was achieved by orally gavaging the mice (8-10 weeks of age) with a cocktail of broad-spectrum antibiotics at intervals of 12h for seven days. The antibiotic cocktail contained ampicillin (100 mg/kg), vancomycin (50 mg/kg), neomycin (100 mg/kg) and metronidazole (100 mg/kg). To prevent opportunistic infections, an antifungal agent amphotericin-B (1 mg/kg) was added as previously described (1, 2). This cocktail was prepared fresh every three days and the twice-daily treatment was given using a gavage volume of 10 ml/kg body weight without sedation (1, 2). The mice assigned to the vehicle/control group received a similar volume of water. The body weight and water consumption for each mouse was recorded throughout the treatment. On day eight, the mice were euthanized, and uteri, blood, and peritoneal fluid were collected. Weights of spleen and cecum were recorded, and the Peyer’s patches were counted.

**Quantitative PCR:** Bacterial DNA isolated from feces of control and microbiota-depleted mice (n=5) was amplified with the following phylum-specific primers (3). Pan-Bacteria, 5′-GCAGGCCTAACACATGCAAGTC-3′ and 5′-CTGCTGCCTCCCGTAGGAGT-3′; Bacteroidetes, 5′-CRAACAGGATTAGATACCCT-3′ and 5′-GGTAAGGTTCCTCGCGTAT-3′; Firmicutes, 5′-TGAAACTYAAAGGAATTGACG-3′ and 5′-ACCATGCACCACCTGTC-3′; Gamma-Proteobacteria, 5′-TCGTCAGCTCGTGTYGTGA-3′ and 5′-CGTAAGGGCCATGATG-3′). The 20 µl PCR reactions contained 4 μl of 10 ng/μl template DNA, 10 μl of 2X Sso Advanced SYBR Green reaction mix (Bio-Rad, Hercules, CA), 0.5 μl of each 10 μM primer, and 5 μl of nuclease-free water. Quantitative PCR was performed with the C1000 Touch Thermocycler (Bio-Rad) and the following conditions: 50 °C for 2 min, 95 °C for 10 min, 45 cycles at 95 °C for 15 s and 61.5 °C for 1 min, followed by a dissociation stage at 65 °C for 31s and cycles of 5s starting at 65 °C, raising 0.5 °C per cycle, to obtain melting curves for specificity analysis. CFX Manager TM software 3.1 (Bio-Rad) was used to obtain Cq values (3). The relative abundances of Bacteroidetes, Firmicutes, and Gammaproteobacteria were calculated after normalizing Cq for each phylum with Cq of Pan-Bacteria.

**Serum 17-β Estradiol level analysis:** The serum level of Serum 17-β Estradiol (E2) (Cat. No. ADI-900-008, Enzo life sciences) was quantified by using ELISA kit as per the instruction provided by manufacturer This 17-β Estradiol ELISA kit is validated and widely used (4, 5).

**IL-1β enzyme-linked immunosorbent assay:** The concentration of IL-1β protein in 50 µl of peritoneal fluid was measured by performing a sandwich immuno-assay (KMC0011, Invitrogen, Life Technologies) as specified in the manufacturer’s protocol. This IL-1β ELISA kit is validated and widely used (6). The concentration of IL-1β was deduced from a standard curve.

**Autologous surgical endometriosis model:** Endometriosis was surgically induced in 10-week-old estrus stage mice by auto-transplanting uterine tissue as previously described (7, 8). Briefly, tissue from one uterine horn from each mouse was excised, cleared of fat tissue, and was cut open longitudinally. Next, using a dermal biopsy punch, a 3 mm^3^ endometrial fragment was isolated from this tissue and grafted on to the peritoneal wall (endometrium facing the peritoneum) in the same mouse through a midline incision (8-10). Sham mice were subjected to a similar surgical procedure of peritoneal wall suture without an endometrial fragment. After 21 days, mice were euthanized, and the following samples were collected: fecal samples and endometriotic lesions. Endometriotic lesion volumes (mm^3^) were measured with a Vernier Caliper.

**Heterologous induction/injection model of endometriosis:** Donor groups of mice were given estradiol benzoate (100 μg/kg) subcutaneously on day -7 (11, 12). At day 0, uteri from donor mice were removed and endometrial tissue was finely chopped into fragments of diameter no greater than 1 mm in sterile saline suspensions. These endometrial suspensions from one donor mouse were then injected into two recipient mice intraperitoneally (13) (14). Precisely, each recipient mouse was challenged with 0.4 mL of endometrial suspension (15). Upon 21 days, mice were sacrificed and endometriotic lesions were carefully excised from the abdominal cavity. The number, weight, and volume of all lesions were used to evaluate the extent of endometriosis in each mouse (11, 12).

**Treatment of mice with Quinic acid:** From day 1 to day 14 after induction of endometriosis, mice were daily orally gavaged with Quinic acid (Cat. No. 138622, Sigma-Aldrich, St. Louis, MO, USA) at 5 mg/kg concentration. Previously, Quinic acid was tested from 2 mg/kg to 20 mg/kg in mice (16, 17). As Quinic acid is water soluble, a similar amount of water was administered as a vehicle. After 14 days, mice were euthanized to collect endometriotic lesions from the abdominal cavity. The total number of lesions, weight, and volume of all lesions per mouse were carefully calculated.

**Fecal microbiota transplantation:** Fecal pellets from sham and endometriosis mice were collected and frozen at -80 °C immediately until use (8, 18). For transplantation, fecal material from 5 mice was pooled and reconstituted in PBS (1 fecal pellet/100 μl of PBS). After filtering through a 70 μM filter to remove the solid material, 200 μl of this fecal filtrate was orally gavaged in microbiota-depleted mice at days 7 and 14 post endometriosis induction.

**Hematoxylin and Eosin staining:** Tissues were fixed in 4% PFA followed by dehydration and embedding in paraffin. Thin tissue sections (5 μm) were cut (Microtome, Leica Biosystem, Germany) and stained with hematoxylin and eosin as described previously (8).

**Immunofluorescence:** Tissue sections were deparaffinized in xylene and rehydrated in graded ethanol. Antigens were retrieved by boiling in citrate-buffer (Vector Laboratories Inc., CA, USA) following which the sections were blocked in 2.5% goat serum (Vector Laboratories Inc., CA, USA) in PBS for 1 h under dark, humidified conditions. Briefly washed sections were incubated at 4 °C overnight with primary antibodies: Anti-Ki-67 (Abcam, ab16667), Anti-CD31 (CST, #77699S) and Anti- F4/80 (Invitrogen, 13-4801-85) diluted 1:200 in normal goat serum. The sections were washed 3 times with PBS and incubated with appropriate Alexa Fluor 488-conjugated secondary antibodies (1:500, Life Technologies) for 1 h at room temperature. Washed tissue sections were mounted with ProLong Gold Antifade Mountant containing DAPI for nuclear staining (Thermo Scientific, MA, USA).

**Flow cytometry:** Mice peritoneal fluid was collected by injection 1mL of sterile PBS, washed twice with 1x PBS, and stained for the live-dead population with Zombie NIR (BioLegend, 1:2000), incubated 5 minutes at room temperature followed by 5 minutes of centrifugation at 4^o^C. Antibody cocktail prepared in brilliant stain buffer (BD Biosciences) was added to the cell pellet, mixed well, and incubated for 20 minutes in dark at 4^o^C. Cells were stained for anti-mouse TCRβ-BV421(H57-597,1:400), CD4-BV570 (RM4-5, 1:400), CD11b-BV650 (M1/70, 1:400), CD45-AF700 (30-F11, 1:400), CD8a-APC/Fire750 (53-6.7, 1:400), F4/80-Super Bright 436 (BM8, 1:400), CD19-BV750 (6D5, 1:400), CD206-AF647 (C068C2, 1:200), CD86-BV605(GL1,1:200). After washing with FACS buffer, cells were fixed with 4% PFA for 30 minutes at room temperature. After incubation, cells were washed again with FACS buffer, and measured on an Aurora using SpectroFlo V2.2.0.3 (Cytek) 4L V-B-YG-R system. Flow cytometry data were analyzed using FCS Express 7.12.0005.

**Targeted metabolomics analysis of fecal samples and statistical analysis:** The fecal samples from sham and endometriosis group were thawed before extraction, and mouse liver pool was used as Quality Control (QC). 750 µL of Methanol and water (4:1) were mixed with an internal standard (ISTD) mix and added to each test sample and quality control. Metabolites were extracted using the liquid-liquid extraction method described previously (19-21). After partitioning through ice-cold chloroform and water, organic and aqueous layers were carefully transferred into new glass vials. Proteins and lipids were removed from extracted samples using a 3K Amicon-Ultra filter (Millipore Corporation, Billerica, MA). The dried pellets were dissolved into methanol-water (50:50 v/v).

The extracted total metabolites samples were analyzed using 3 methods through high-throughput Liquid Chromatography-Mass Spectrometry (LC-MS/MS) techniques described previously (19, 20). The metabolites were separated through the XBridge Amide HPLC column (3.5 µm, 4.6 x 100 mm, Waters, Milford, MA) in both ESI positive (**Method A**) and negative mode (**Method B**). For ESI positive, mobile phase A and B were 0.1% formic acid in water and acetonitrile, respectively. Gradient flow: 0-3 min 85% B; 3-12 min 30% B, 12-15 min 2% B, 16 min 95% B, followed by re-equilibration till the end of the gradient 23 minute to the initial starting condition of 85% B. Flow rate of the solvents used for the analysis is 0.3 mL/minute. The injection volume was 5 µL. For ESI negative, mobile phase A and B were 20 mM ammonium acetate in water with pH 9.0 and 100% acetonitrile, respectively. Gradient flow: 0-3 min 85% B, 3-12 min 30% B, 12-15 min 2% B, 15-16 min 85% B followed by re-equilibration till the end of the gradient 23 minute to the initial starting condition of 85% B. Flow rate of the solvents used for analysis is 0.3 mL/minute. The injection volume was 10 µL. Thirdly, the metabolites were separated through Luna 3 µM NH2 100 A^0^ HPLC column (Phenomenex, Torrance, CA, USA) in ESI positive mode (**Method C**). The mobile phase, injection volume, and flow rate was the same as **Method B**.

Above mentioned volume of samples was injected and acquired the data via multiple reaction monitoring (MRM) using a 6495 Triple Quadrupole mass spectrometry coupled to an HPLC system (Agilent Technologies, Santa Clara, CA, USA) through Agilent Mass Hunter Software. The details of the LC-MS methods are discussed in Gohlke et al. 2019 (19). The acquired data were analyzed and integrated of each peak using Agilent Mass Hunter Quantitative Analysis software.

**Cell culture and cell viability assays:** Immortalized Human Endometriotic Epithelial Cells/Luciferase (iHEECs/Luc) cells were grown in DMEM/F12 media containing 10% FBS and supplemented with antibiotics: penicillin (100 U/mL), streptomycin (100 mg/mL), and Amphotericin-B (2.5 mg/mL). The cells were incubated at 37 °C under humidified conditions with 5% CO_2_ and the media was replenished on every alternate day (22). Cell viability was determined by performing the MTT assay (Promega) according to the manufacturer's instructions and as described previously (22). Briefly, iHEECs/Luc were seeded in 96-well plates at a density of 5×10^3^ cells per well. After 24 hours, cells were treated with Vehicle, and different concentration of Quinic acid, Cytosine, 1-Methyl-Histidine, Ng, NG-Dimethyl L-Arginine, 2-Amino heptanoic acid and N-Acetyl Aspartic acid (Sigma-Aldrich, St. Louis, MO, USA) and relative viability rates were evaluated for indicated time points (**Fig. 7C** and **Fig. S3B-F**). For this, 15 µL of MTS dye was added to each well and plates were incubated at 37 °C for another 2 hours. Finally, 100 µL of solubilization solution was added and absorbance recorded at test wavelength of 570 nm and reference wavelength of 650 nm. Each treatment group was independently assayed three times with three to five technical replicates.

**Supplementary References**

1. Reikvam DH, Erofeev A, Sandvik A, Grcic V, Jahnsen FL, Gaustad P, et al. Depletion of murine intestinal microbiota: effects on gut mucosa and epithelial gene expression. PLoS One. 2011;6(3):e17996.

2. Zarrinpar A, Chaix A, Xu ZZ, Chang MW, Marotz CA, Saghatelian A, et al. Antibiotic-induced microbiome depletion alters metabolic homeostasis by affecting gut signaling and colonic metabolism. Nat Commun. 2018;9(1):2872.

3. Trompette A, Gollwitzer ES, Yadava K, Sichelstiel AK, Sprenger N, Ngom-Bru C, et al. Gut microbiota metabolism of dietary fiber influences allergic airway disease and hematopoiesis. Nat Med. 2014;20(2):159-66.

4. Yoon HJ, Lee YJ, Baek S, Chung YS, Kim DH, Lee JH, et al. Hormone autocrination by vascularized hydrogel delivery of ovary spheroids to rescue ovarian dysfunctions. Sci Adv. 2021;7(18).

5. Zhang JH, Tasaki T, Tsukamoto M, Wang KY, Azuma K. Deficiency of Wnt10a causes female infertility via the beta-catenin/Cyp19a1 pathway in mice. Int J Med Sci. 2022;19(4):701-10.

6. Zaheer A, Zaheer S, Sahu SK, Knight S, Khosravi H, Mathur SN, et al. A novel role of glia maturation factor: induction of granulocyte-macrophage colony-stimulating factor and pro-inflammatory cytokines. J Neurochem. 2007;101(2):364-76.

7. Pelch KE, Sharpe-Timms KL, Nagel SC. Mouse model of surgically-induced endometriosis by auto-transplantation of uterine tissue. J Vis Exp. 2012(59):e3396.

8. Chadchan SB, Cheng M, Parnell LA, Yin Y, Schriefer A, Mysorekar IU, et al. Antibiotic therapy with metronidazole reduces endometriosis disease progression in mice: a potential role for gut microbiota. Hum Reprod. 2019;34(6):1106-16.

9. Zhao Y, Chen Y, Kuang Y, Bagchi MK, Taylor RN, Katzenellenbogen JA, et al. Multiple Beneficial Roles of Repressor of Estrogen Receptor Activity (REA) in Suppressing the Progression of Endometriosis. Endocrinology. 2016;157(2):900-12.

10. Zhao Y, Gong P, Chen Y, Nwachukwu JC, Srinivasan S, Ko C, et al. Dual suppression of estrogenic and inflammatory activities for targeting of endometriosis. Sci Transl Med. 2015;7(271):271ra9.

11. Yuan M, Li D, Zhang Z, Sun H, An M, Wang G. Endometriosis induces gut microbiota alterations in mice. Hum Reprod. 2018.

12. Bacci M, Capobianco A, Monno A, Cottone L, Di Puppo F, Camisa B, et al. Macrophages are alternatively activated in patients with endometriosis and required for growth and vascularization of lesions in a mouse model of disease. Am J Pathol. 2009;175(2):547-56.

13. Yuan M, Li D, An M, Li Q, Zhang L, Wang G. Rediscovering peritoneal macrophages in a murine endometriosis model. Hum Reprod. 2017;32(1):94-102.

14. Long Q, Liu X, Guo SW. Surgery accelerates the development of endometriosis in mice. Am J Obstet Gynecol. 2016;215(3):320 e1- e15.

15. Somigliana E, Vigano P, Rossi G, Carinelli S, Vignali M, Panina-Bordignon P. Endometrial ability to implant in ectopic sites can be prevented by interleukin-12 in a murine model of endometriosis. Hum Reprod. 1999;14(12):2944-50.

16. Jang G, Lee S, Hong J, Park B, Kim D, Kim C. Anti-Inflammatory Effect of 4,5-Dicaffeoylquinic Acid on RAW264.7 Cells and a Rat Model of Inflammation. Nutrients. 2021;13(10).

17. Lin H, Wu Y, Chen J, Huang S, Wang Y. (-)-4-O-(4-O-beta-D-glucopyranosylcaffeoyl) Quinic Acid Inhibits the Function of Myeloid-Derived Suppressor Cells to Enhance the Efficacy of Anti-PD1 against Colon Cancer. Pharm Res. 2018;35(9):183.

18. Hintze KJ, Cox JE, Rompato G, Benninghoff AD, Ward RE, Broadbent J, et al. Broad scope method for creating humanized animal models for animal health and disease research through antibiotic treatment and human fecal transfer. Gut Microbes. 2014;5(2):183-91.

19. Gohlke JH, Lloyd SM, Basu S, Putluri V, Vareed SK, Rasaily U, et al. Methionine-Homocysteine Pathway in African-American Prostate Cancer. JNCI Cancer Spectr. 2019;3(2):pkz019.

20. Putluri N, Shojaie A, Vasu VT, Vareed SK, Nalluri S, Putluri V, et al. Metabolomic profiling reveals potential markers and bioprocesses altered in bladder cancer progression. Cancer Res. 2011;71(24):7376-86.

21. Vantaku V, Dong J, Ambati CR, Perera D, Donepudi SR, Amara CS, et al. Multi-omics Integration Analysis Robustly Predicts High-Grade Patient Survival and Identifies CPT1B Effect on Fatty Acid Metabolism in Bladder Cancer. Clin Cancer Res. 2019;25(12):3689-701.

22. Chadchan SB, Popli P, Ambati CR, Tycksen E, Han SJ, Bulun SE, et al. Gut microbiota-derived short-chain fatty acids protect against the progression of endometriosis. Life Sci Alliance. 2021;4(12).

**Supplementary Figure legends:**

**Fig. S1:** **Schematic of experimental timeline and procedure.** Schematic of experimental procedure and timeline for **A** suture based, and **B** injection-based endometriosis mouse model.

**Fig. S2:** **Fecal microbiota transfer (FMT) restores the endometriotic lesion proliferation, angiogenesis, and inflammation.** **A-B** Representative images of ectopic lesions from the indicated groups stained with **A** Hematoxylin & Eosin, blue arrows indicate epithelium, and **B** anti-Ki-67 antibody. **C-D** Percentages of Ki-67-positive cells in endometriotic lesion **C** epithelium and **D** stroma expressed in terms of percent positive cells. **E-F** Representative images of ectopic lesions from the indicated treatment groups stained with **E** anti-CD31 antibody and **F** anti-F4/80 antibody. E, epithelium; S, stroma. White arrows indicate positive cells. Data are presented as mean ± SE (n=5), **P* < 0.05, ***P* < 0.01, and ns, non-significant.

**Fig. S3:** **Differentially present metabolites regulate cell viability.** **A** Differentially present metabolites Cytosine, 1-Methyl-histidine, Ng,NG-dimethyl L-arginine, 2-Amino heptanoic acid and N-Acetyl Aspartic acid in feces of mice of sham and endometriosis. **B-F** MTT cell viability assays of iHEECs/Luc treated with different concentration of metabolites, Cytosine, 5-Methyl Histidine, 5-Methyl Arginine, 2-Amino Hepatonic acid, and N-Acetyl Aspartic acid for indicated time points. Results are shown as mean ± SE (n=3) and experiment repeated three times. **P* < 0.05 and ns, non-significant.
