# Supplementary figures and images for: Gut microbiota and microbiota-derived metabolites promotes endometriosis
Source: Cell Death Discov. 2023 Jan 25;9:28. doi: 10.1038/s41420-023-01309-0 (PMC9873805; doi:10.1038/s41420-023-01309-0)

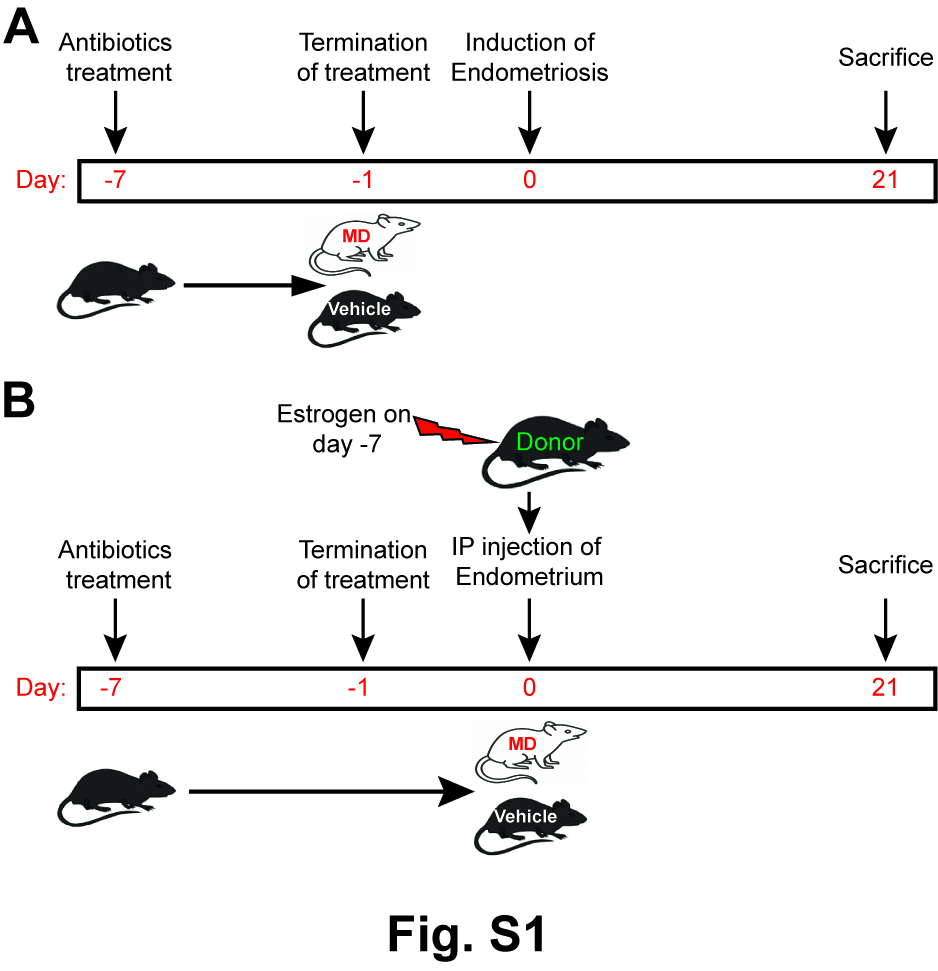

Supplement: Supplementary file 2 — Figure S1 [file 41420_2023_1309_MOESM2_ESM.tif]

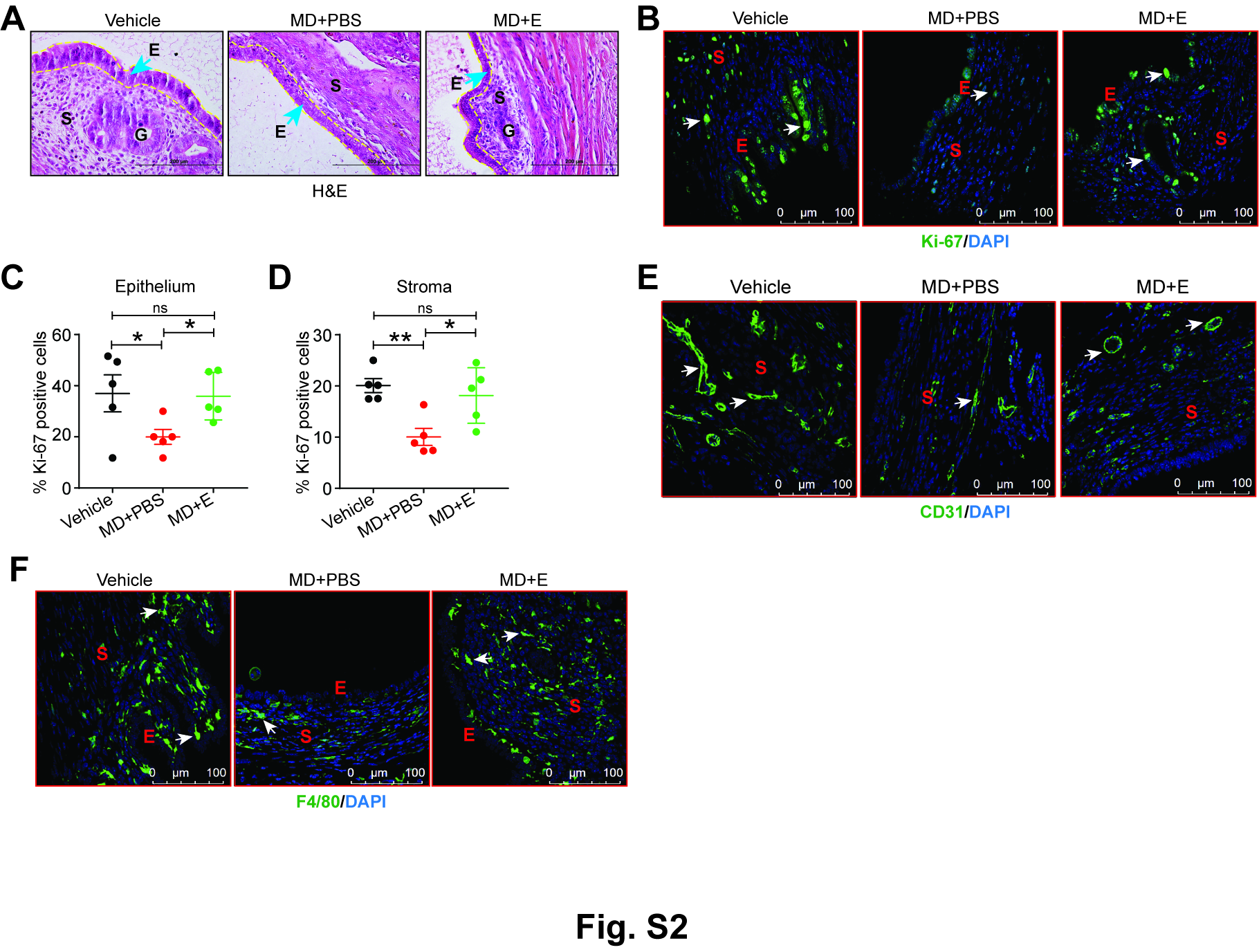

Supplement: Supplementary file 3 — Figure S2 [file 41420_2023_1309_MOESM3_ESM.tif]

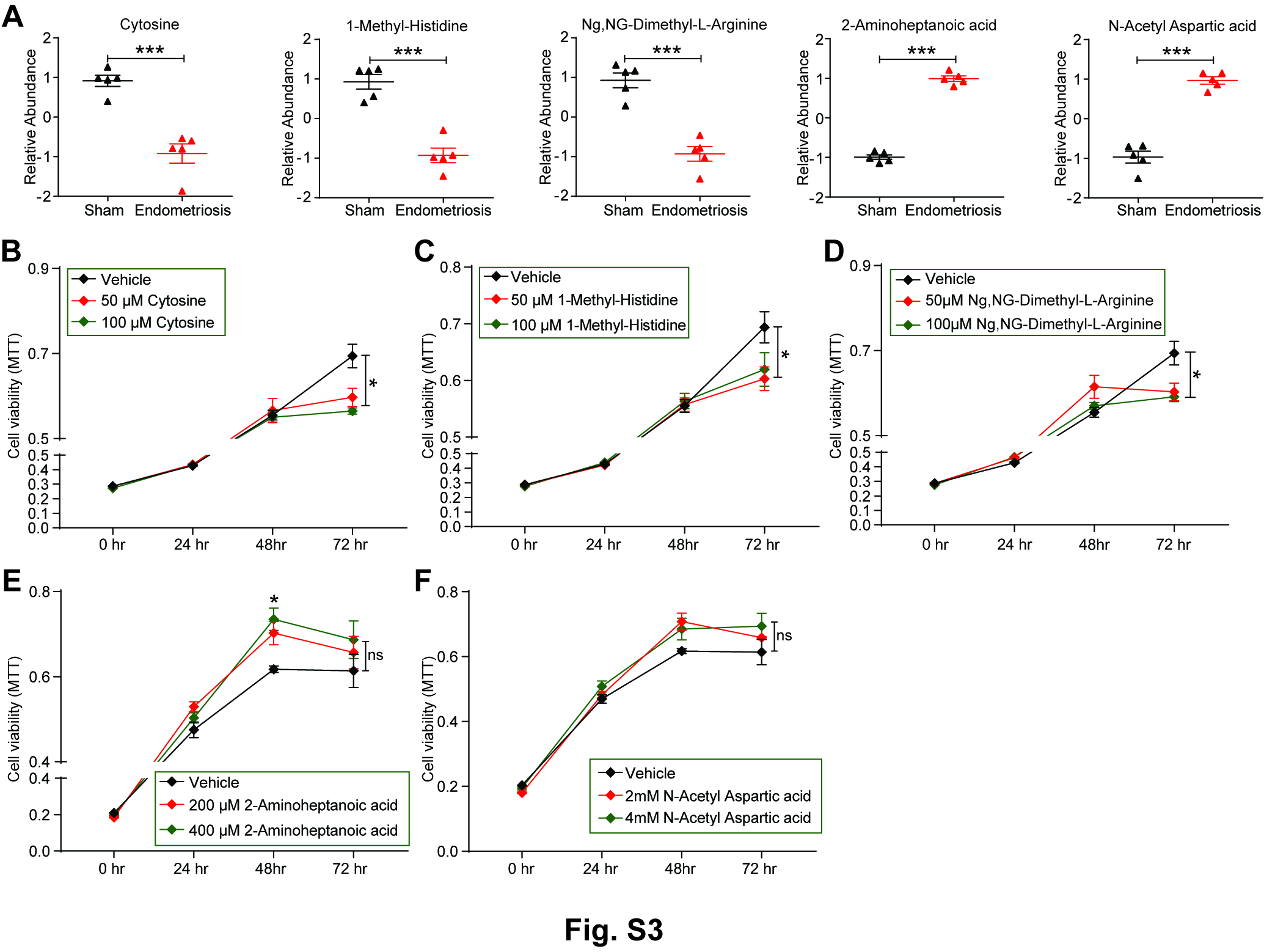

Supplement: Supplementary file 4 — Figure S3 [file 41420_2023_1309_MOESM4_ESM.tif]
